# lncRNA Sequencing Reveals Neurodegeneration-associated FUS Mutations Alter Transcriptional Landscape of iPS Cells That Persists In Motor Neurons

**DOI:** 10.21203/rs.3.rs-3112246/v1

**Published:** 2023-06-27

**Authors:** Vincent E. Provasek, Manohar Kodavati, Wenting Guo, Haibo Wang, Istvan Boldogh, Ludo Van Den Bosch, Gavin Britz, Muralidhar Hegde

**Affiliations:** 1 Division of DNA Repair Research within the Center for Neuroregeneration, Department of Neurosurgery, Houston Methodist Research Institute, Houston, TX 77030, USA.; 2 School of Medicine, Texas A&M University, College Station, TX 77843, USA.; 3 Department of Microbiology and Immunology, University of Texas Medical Branch, Galveston, TX 77555, USA.; 4 KU Leuven-Department of Neurosciences, Experimental Neurology and Leuven Brain Institute (LBI), Leuven, 3000, Belgium.; 5 Stem Cell Institute, Department of Development and Regeneration, KU Leuven, Leuven, Belgium; 6 Department of Neurosurgery, Houston Methodist Research Institute, Houston, TX 77030, USA.; 7 Weill Cornell Medical College, New York, NY 10065, USA.

**Keywords:** Fused-in Sarcoma (FUS), neurodegenerative disorders, induced pluripotent stem cells (iPSCs), long non-coding RNAs (lncRNAs), RNA sequencing

## Abstract

*Fused-in Sarcoma* (*FUS*) gene mutations have been implicated in amyotrophic lateral sclerosis (ALS). This study aimed to investigate the impact of FUS mutations (R521H and P525L) on the transcriptome of induced pluripotent stem cells (iPSCs) and iPSC-derived motor neurons (iMNs). Using RNA sequencing (RNA Seq), we characterized differentially expressed genes (DEGs), differentially expressed lncRNAs (DELs), and subsequently predicted lncRNA-mRNA target pairs (TAR pairs). Our results show that FUS mutations significantly altered expression profiles of mRNAs and lncRNAs in iPSCs. We identified key differentially regulated TAR pairs, including LMO3, TMEM132D, ERMN, GPR149, CRACD, and ZNF404 in mutant FUS iPSCs. We performed reverse transcription PCR (RT-PCR) validation in iPSCs and iMNs. Validation confirmed RNA-Seq findings and suggested that mutant FUS-induced transcriptional alterations persisted from iPSCs into differentiated iMNs. Functional enrichment analyses of DEGs indicated pathways associated with neuronal development and carcinogenesis that were likely altered by FUS mutations. Ingenuity Pathway Analysis (IPA) and GO network analysis of lncRNA-targeted mRNAs indicated associations related to RNA metabolism, lncRNA regulation, and DNA damage repair. Our findings provide insights into the molecular mechanisms underlying the pathophysiology of ALS-associated FUS mutations and suggest potential therapeutic targets for the treatment of ALS.

## Introduction

Neurodegenerative disorders, such as amyotrophic lateral sclerosis (ALS) pose significant challenges to our understanding of the molecular mechanisms that drive neuronal dysfunction and degeneration. While 90% of ALS is sporadic (sALS), the remaining 10% of patients suffer from the familial variant of the disease (fALS). One of the mutated genes responsible for fALS, the *Fused-in Sarcoma (FUS)* has been identified as an important contributor to the pathogenesis of these debilitating diseases ^1^. FUS is an RNA/DNA-binding protein that plays a crucial role in various aspects of RNA metabolism, including transcription, splicing, and transport, in addition to its role in DNA repair^2,3^. Dysregulation of FUS function due to mutations has been implicated in the pathogenesis of ALS and FTD, although the precise molecular events leading to neurodegeneration remain unclear.

FUS mutations typically disrupt the nuclear localization signal (NLS) of the protein and can affect its RNA-binding capacity. Key mutations in the FUS gene, such as R521H and P525L, are located within the NLS domain and have been shown to promote the mislocalization of FUS from the nucleus to the cytoplasm of motor neurons and glial cells in ALS and FTD patients, leading to its aggregation^4^. This aberrant FUS localization results in both loss of its normal function in the nucleus and gain of toxic properties in the cytoplasm, suggesting that FUS mislocalization and aggregation plays a central role in disease pathogenesis^5^.

FUS-associated neurodegeneration is thought to be driven by a combination of loss of nuclear function and cytoplasmic toxicity^6,7^. The loss of FUS function in the nucleus may lead to impaired RNA processing and transcriptional regulation as well as defective DNA repair, thereby affecting neuronal survival and function^2,8–12^. On the other hand, the accumulation of cytoplasmic FUS aggregates may disrupt cellular homeostasis by impairing the function of other RNA-binding proteins and sequestering essential cellular components^7,13^. This dual mechanism, involving both loss of nuclear function and gain of cytoplasmic toxicity, may contribute to the complex pathophysiology of ALS and FTD. Previous studies have implicated alterations in RNA metabolism, stress granule dynamics, and DNA damage repair pathways in FUS-associated neurodegeneration^14–16^. However, a comprehensive understanding of the transcriptional landscape and the affected pathways in the context of FUS mutations is still lacking.

LncRNAs play a significant role in regulating gene expression, protein activity, and chromatin structure. They can interact with DNA, RNA, and proteins and have been shown to regulate various biological processes, including development, differentiation, and diseases^17,18^. Altered RNA metabolism regulation has been identified as a critical factor in the pathogenesis of ALS, with many familial mutations associated with disease pathology occurring in DNA/RNA-binding proteins involved in RNA metabolism, such as FUS, TDP-43, SOD1, hnRNP proteins, and others^19^. In particular, FUS has been shown to interact with a wide variety of RNAs, including mRNAs, miRNAs, and lncRNAs, and to play a global role in lncRNA regulation^20^. FUS has also been shown to be important for the localization of lncRNAs to specific subcellular compartments, such as the nucleus or cytoplasm, with significant consequences for their function. The lncRNA nuclear paraspeckle assembly transcript 1 (NEAT1) is one such example, where FUS has been shown to bind to a specific region of NEAT1 and assist in localizing it to nuclear paraspeckles^5,21^. Furthermore, FUS is necessary for the formation of paraspeckles in some cell types, suggesting that FUS-NEAT1 interactions play a role in the regulation of this subnuclear structure. Interestingly, proteins enriched in the pool of proteins affected by ALS-causative mutations are also found in paraspeckles.

Here, we employed RNA-Seq to examine the effects of neurodegeneration-linked FUS mutations (R521H and P525L) on the transcriptional profiles of iPSCs and assessed whether these changes persisted in differentiated motor neurons. We analyzed the expression profiles of both mRNAs and long non-coding RNAs (lncRNAs) in control and FUS-mutant iPSCs, highlighting the role of lncRNAs in modulating gene expression. We identified differentially expressed genes (DEGs) and differentially expressed lncRNAs (DELs) in FUS-mutant iPSCs and predicted lncRNA-mRNA target pairs (TAR pairs). Moreover, we identified significant biological processes involving RNA metabolism, lncRNA regulation, and DNA damage repair through Ingenuity Pathway Analysis (IPA) and GO network analysis of lncRNA-targeted mRNAs.

This study thus uncovers novel aspects of the molecular mechanisms underlying the pathophysiology of FUS mutations in neurodegenerative diseases and identifies potential therapeutic targets for treating ALS and FTD.

## Results

### Results of sequencing and characteristics of transcripts

**Fig 1.**
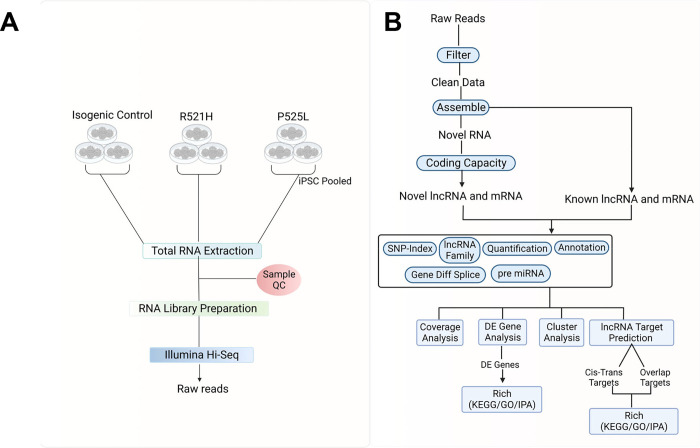
Overview of the study’s experimental approach: **A)** Cultured patient-derived induced pluripotent stem cells (iPSCs) harboring Fused-in Sarcoma (FUS) gene mutations R521H and P525L were utilized. Total RNA was extracted for sequencing after pooling three distinct cultures. **B)** The bioinformatics analysis workflow employed in the study.

The overall design of this study is depicted in Figure 1. Human derived iPSCs were grown in triplicate under standard conditions before pooling and total RNA isolation. After verifying the quality of the extracted RNA, Illumina Hi-Seq libraries were prepared. Short read sequencing was used to analyze the transcript`tomes of iPSCs derived from ALS patients carrying the FUS P525L or FUS R521H mutations and compared to cells derived from a healthy control patient. A total of 106,975,508 reads were collected from control samples and 108,244,484 and 92,527,778 reads collected from the P525L and R521H mutants, respectively (Table 1). These data were then filtered to remove low-quality reads, adaptor sequences and general quality control which yielded an acceptable total of 99,081,102 clean reads for the control, and 99,842,418 and 85,427,768 clean reads for the P525L and R521H mutants, respectively. We observed an acceptable mapping rate of these clean reads to the reference transcriptome (GRh38) of 92.25% for control and 93.48% and 94.21% for P525L and R521H, respectively. Over 80% of all reads mapped to a single location in the reference transcriptome, indicating the data was acceptable for accurate differential gene expression analysis. Additional general characteristics of mapped reads are summarized in Figures S1 and S2.

**Fig 2.**
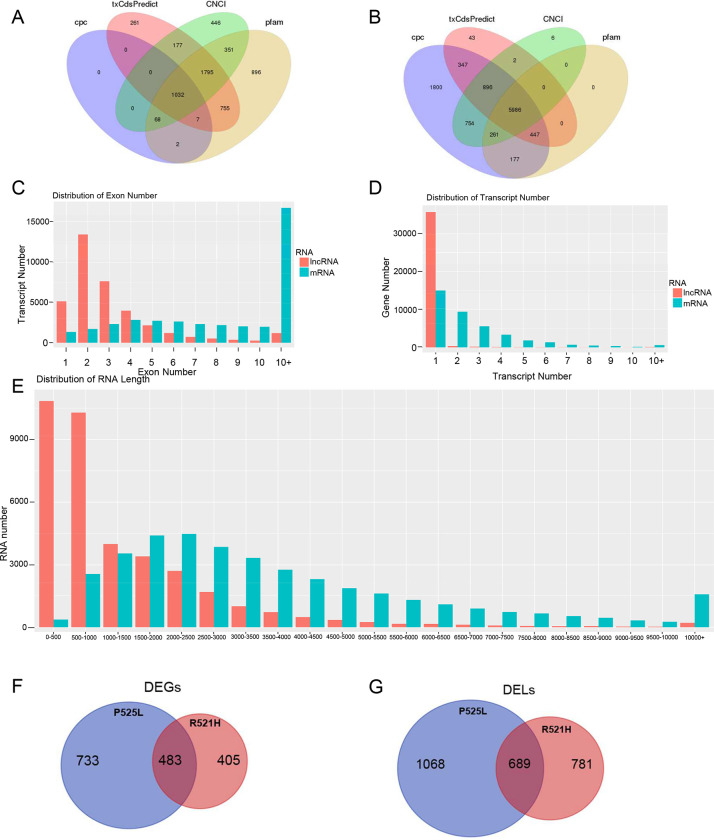
Specific characteristics of human iPS cell derived mRNAs and lncRNAs. **A)** Exon number analysis in transcripts, including mRNA and lncRNA; X-axis: number of exons, Y-axis: number of transcripts, color: RNA classification. **B)** Distribution of the transcripts number for genes, including mRNA and lncRNA, X-axis: number of transcripts, Y-axis: number of genes, color: RNA classification. **C)** The statistics figure of RNA length, including mRNA and lncRNA, X-axis: RNA length, Y-axis: number of transcripts, color: RNA classification. **D)** Venn diagram of predicted result mRNA. Different colors represent different prediction methods performed on the same samples. **E)** Venn diagram of predicted result mRNA. Different colors represent different prediction methods performed on the same samples. result mRNA. Different colors represent different prediction methods performed on the same samples. **F**) Venn diagram of significant differentially expressed mRNA genes (DEGs) in FUS mutants P525L and R521H over WT. **G**) Venn diagram of significant differentially expressed lncRNAs (DELs) in FUS mutants P525L and R521H over WT.

Identification of lncRNAs was conducted by predicting the coding ability of the uniquely mapped reads to distinguish them from mRNA transcripts (Figure 2 A–B). Three predictive software tools were used to score the coding ability of the transcripts using a reference database (pfam). Transcripts mapping to the pfam database were classified as mRNA while the remainder were classified as lncRNAs. The lncRNA dataset used for downstream analysis was constructed using only the lncRNAs commonly identified by all three tools. This analysis step identified 13,102 total lncRNAs in the control sample, while a total of 13,232 and 12,795 lncRNAs were found in P525L and R521H mutants, respectively. Interestingly, this analysis discovered over one thousand novel lncRNAs across both mutants. This analysis also identified over nineteen-thousand known mRNAs with over four-thousand of them identified as novel across all samples (Table 2).

Additional characteristics of the mapped transcripts are shown in Fig. 2. As expected, analysis of the distribution of exon number across the transcripts revealed lncRNAs mostly contained two exons while mRNAs contained more than ten (Figure 2 C–D). Similarly, transcripts with a length of 2–2.5kb made up the majority of mRNAs while lncRNAs were comprised mostly of transcripts between 0–500bp (Figure 2E). Additionally, when we analyzed the number of DEGs and DELs between each comparison group, we discovered FUS-P525L mutant cells contained 58% more DEGs and 31% more DELs relative to controls (Figure 2 F–G).

### Differential expression analysis

**Fig 3.**
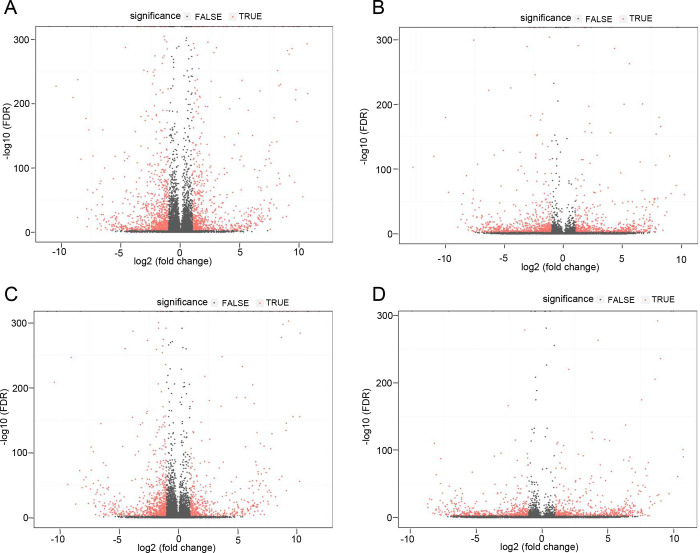
Expression profiles of distinct RNAs in human iPS cells carrying neurodegeneration-associated mutations in FUS. Volcano plots depict log2 fold change in uniquely mapped mRNA and lncRNAs from each pooled sample of control and FUS-mutant human iPS cells. **A)** Control vs P252L, mRNA. **B)** Control vs P525L, lncRNA. **C)** Control vs R521H, mRNA. **D)** Control vs R521H, lncRNA.

To calculate differential expression analysis of genes and transcripts, Bowtie2^22^ was used to align clean reads to the reference sequence and then RSEM^23^ was used to calculate gene and transcript expression levels. Subsequently, the PossionDis^24^ tool (PossionDisFoldChange>=2.00 and FDR<=0.001) was used to analyze the significance of DELs and DEGs between the samples SA (control) and samples SB (FUS-P525L) and SC (FUS-R521H). The analysis of DEGs and DELs are depicted in Figure 3 A–D. In total, 1,734 significantly differentially expressed mRNAs and 1,239 lncRNAs were identified in the control as compared to the P525L samples while 1,317 mRNAs and 1,041 lncRNAs were detected in the control as compared to the R521H samples. Interestingly, there was a significant overlap between the most upregulated genes in the comparisons between the control and R521H. Out of the top ten most upregulated and downregulated genes, comparisons between controls and each mutant shared six commonly identified targets. These included RPS4y1, DDX3Y, MXLOC_037825, EIF1AY, RPL17-C18orf32, and n379185. Evaluation of the most downregulated genes revealed slightly less concordance, with each comparison between control and mutant sharing only three of the top ten most downregulated genes. These included MXLOC_016157, MAGEA12, and LXLOC_037100. With respect to the total number of differentially expressed genes, the control vs P525L and control vs R521H comparisons yielded 711 and 593 upregulated targets, respectively. The same analysis of downregulated genes revealed 839 and 637 targets for each control vs P525L and control vs R521H comparisons, respectively. Overall, these findings suggest that while each of the two different mutations in the same FUS protein exerted similar effects on the transcriptional landscape of iPSCs, there are important distinctions to consider in future work. A full listing of differentially regulated genes from each comparison are listed in Table S2 and S3.

### Identification of differentially regulated lncRNA-mRNA target pairs

**Fig 4.**
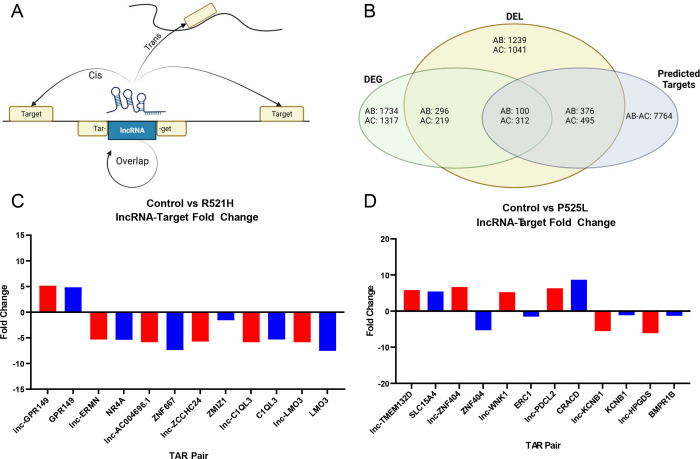
RNA-Seq reveals key differentially regulated lncRNA-mRNA target pairs in FUS mutant human iPS cells. **A)** Schematic illustrating how lncRNAs may target mRNAs in a cis manner (i.e., mRNAs within 20kB of the lncRNA) or trans (i.e., predicted based on calculated binding energy between the TAR pair). **B)** Venn Diagram outlining results of manual annotation of raw data sets. A total of 1734 and 1317 well annotated DEGs and 1239 and 1042 DELs were identified and cross referenced against a total of 7764 predicted TAR pairs. Data sets were further refined to reveal 100 and 312 TAR pairs wherein each lncRNA and mRNA target were differentially regulated by 5-fold or greater. **C-D)** The top six most differentially regulated TAR pairs were selected and log2 fold expression visualized. The lncRNA fold expression vs control shown in red and fold expression of each lncRNA target mRNA shown in blue. **DEG** indicates Differentially Expressed Genes referencing mRNAs; **DEL** is Differentially Expressed lncRNAs.

One of the mechanisms by which lncRNAs can alter gene expression is through cis- or transacting effects with target mRNAs (Figure 4A). To this end, we questioned whether any relationship existed between the DEGs and DELs identified between the control and FUS mutant iPSCs. To accomplish this, we cross-referenced datasets containing lists of DEGs and DELs against a list of software generated predictions of lncRNA-mRNA target pairs (TarPairs). The results of this analysis are summarized in Figure 4B. We refined the list of 7,764 TarPairs by filtering those pairs whose members were differentially regulated by a factor of two-fold or greater in either direction. This filtering step resulted in the identification of 100 significantly regulated TarPairs between the control and the P525L mutant, and 312 between the control and the R521H mutant. Figure 4 C–D show selected TarPairs exhibiting at least a five-fold expression difference between control vs P525L and control vs R521H, respectively. The selected TarPairs for the control vs P525L included lnc-GPR149, GPR149, lncERMN, NR4A, lnc-AC004696.1, ZNF667, lnc-ZCCHC24, ZMIZ1, lnc-C1QL3, lnc-LMO3, and LMO3. The selected TarPairs for the control vs R521H included lnc-TMTM132D, SLC15A4, lnc-ZNF404, ZNF404, lnc-WNK, ERC1, lnc-TSPY2, TBL1Y, lnc-KCNB1, lncHPGDS, and BMPR1B. In the majority of cases, mRNA target expression was congruent with its putative interacting lncRNA. In other cases, such as the ZNF404 TarPair, the expression was inversed.

### RT-PCR confirmation of TarPairs in iPSC and motor neuron cell cultures

**Fig 5.**
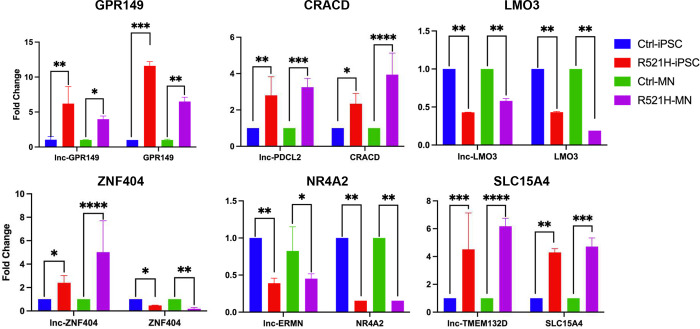
RT-PCR Validation of RNA-Seq Results. Select DELs (black) and predicted TAR pairs (pink) were validated by RT-PCR for human-derived iPSCs and induced motor neuron cultures differentiated from the same iPSC lines. Validation experiments confirmed RNA-Seq findings and suggested that mutant FUS induced transcriptional alteration persist into the differentiated motor neuron stage. iPSC, induced pluripotent stem cell; MN, motor neuron.

To validate our sequencing results, six TarPairs were selected for RT-PCR analysis for their strong differential expression. Validation experiments were conducted using RNA samples extracted from the same iPSC cultures used for sequencing. As shown in Figure 5, expression patterns of the DELs and their predicted target DEGs remained consistent with our sequencing data. Given the disease relevance of FUS P525L and R521H mutations for ALS pathogenesis, we questioned whether these effects persisted in motor neurons. To answer this question, we removed a subset of iPSCs used for RNA-Seq and RT-PCR analysis and differentiated them into terminally differentiated motor neurons using methods previously described^25^. RNA samples from these motor neurons were then evaluated by RT-PCR and the results are shown in Figure 5. Interestingly, each of the selected TarPairs maintained the differential expression pattern observed in their iPSC precursor, albeit to different degrees. Moreover, repeat experiments using isogenic control lines showed reversal of the mutant effect (Figure S3). Taken together, these data suggest FUS mutant associated alterations in lncRNA and mRNA expression are unique and persistent across stages of cell development. All RT-PCR reactions were conducted in triplicate. Statistical significance was determined using Student’s t-test where p<0.05 was considered significant. Primers used for RT-PCR confirmation are listed in Table S1.

### Functional enrichment analysis of mRNAs co-expressed with lncRNAs

**Fig 6.**
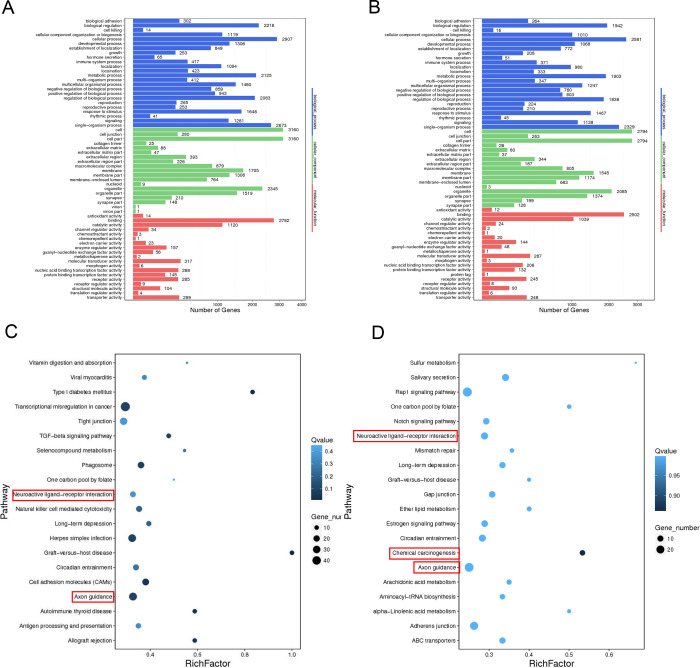
Enrichment Analysis of Differentially Expressed Genes. **A-B)** Gene Ontology (GO) enrichment analysis of differentially expressed genes between Control vs R521H and P525L, respectively. X-axis: number of genes, Y-axis: GO entry, color: GO classification. **C-D)** KEGG enrichment analysis of differentially expressed genes between Control vs R521H and P525L, respectively. Multiple pathway analyses indicate pathways associated with neuronal development and carcinogenesis are likely altered by FUS mutations. X-axis: enrichment factor, Y-axis: pathway, color: p-value, size: number of genes. **E**) The GO analysis results.

**Fig 7.**
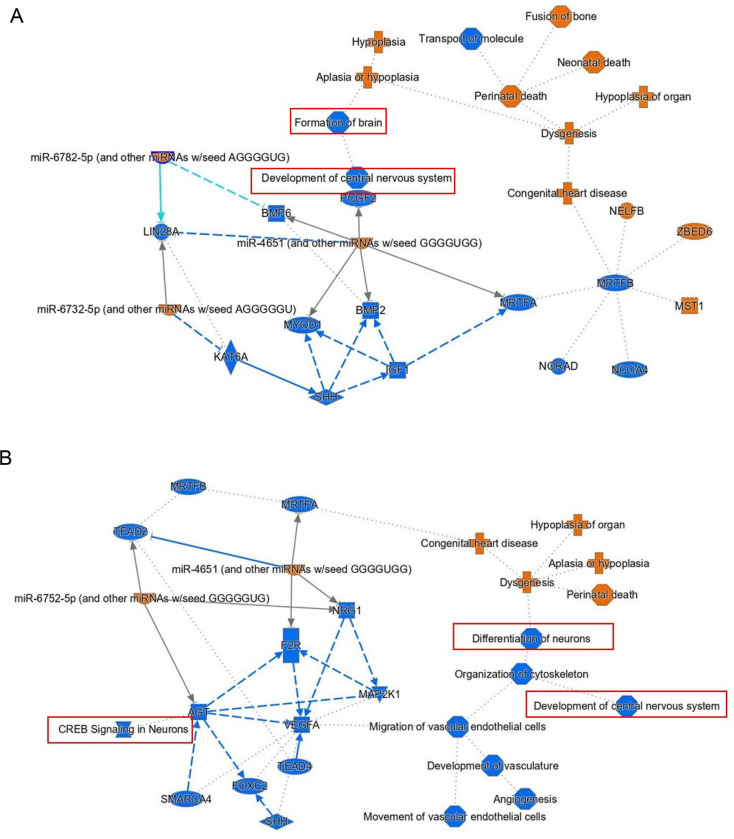
IPA Network Analysis. Ingenuity Pathway Analysis (QIAGEN) of DEGs revealed both inferred and direct affecting network connections involved in development of the CNS and neural signaling (outlined in red). IPA analysis performed on DEGs identified from comparisons between **A)** Control vs P525L, and **B)** Control vs R521H.

**Fig 8.**
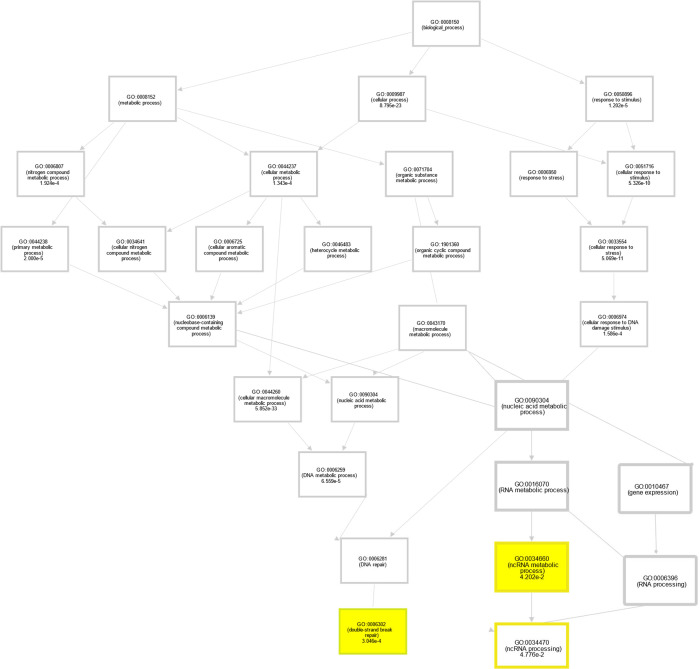
GO network analysis of lncRNA targeted mRNAs. GO network analysis of lncRNA targeted mRNAs indicated significant biological processes associated with RNA metabolism, lncRNA regulation, and DNA damage repair (highlighted in yellow).

Multiple network and functional pathway analysis tools were used to infer functional consequences of the differentially expressed genes observed in FUS mutant iPSCs compared to the control. The potential function of FUS P525L and R521H mutations were studied using gene ontology (GO) annotation and enrichment analysis. For GO enrichment analysis of differentially expressed genes between samples, targets were classified into three general categories of biological processes, molecular function, and cellular component (Figure 6 A–B). Within the biological process class, terms with the greatest number of associated genes found in both mutants included response to stimulus (GO:0050896), regulation of biological process (GO:0048519), cellular process (GO:0009987) and biological regulation (GO:0065007). We also analyzed functional enrichment analysis using the Kyoto Encyclopedia of Genes and Genomes (KEGG) tool (Figure 6 C–D). Interestingly, we observed that functional enrichment included multiple nervous system related terms including neuroactive ligand-receptor interactions, axon guidance and cell adhesion molecules. Additionally, we observed functional enrichment around transcriptional misregulation in cancer, a function that might be expected given the strong association of FUS with neoplastic pathologies. We observed similar functional enrichment in the control vs R521H comparison. Finally, we complemented these two approaches with Ingenuity Pathway Analysis (QIAGEN) performed on DEGs identified between each mutant comparison (Figure 7 A–B). Despite these analyses being conducted in iPSCs, we again observed network effects associated with the nervous system, including CNS development in the control vs P525L and neuronal differentiation and signaling in the control vs R521H. Finally, we conducted GO analysis using predicted mRNA targets of identified lncRNAs (Figure 8). Given the general function of FUS as a regulator of RNA function, we observed significant GO terms indicating noncoding RNA processing (GO:0006396) and metabolic processes (GO:0034660). Additionally, we observed significant terms DNA damage signaling and repair (GO:0006302).

## Discussion

In this study, we identified differentially expressed genes, lncRNAs, and lncRNA-mRNA target pairs (TAR pairs) in association with R521H and P525L FUS mutations in cultured iPSCs and iMNs derived from patients. Our study demonstrates the impact of neurodegeneration associated FUS mutations R521H and P525L on the transcriptional landscape in cells across differentiation states. By conducting RNA-seq analysis, we characterized the expression profiles of both mRNAs and lncRNAs in control and FUS-mutant iPSCs. Our findings revealed significant changes in the expression profiles of distinct lncRNAs in FUS-mutant iPSCs. Notably, these differentially expressed lncRNAs were correlated with a similar change in the expression of their putative target mRNAs. Unlike conventional RNA-Seq studies that primarily focus on changes in the landscape of mRNA or non-coding RNAs, our study is unique in that we have identified and validated co-expression of predicted lncRNA-mRNA TAR pairs in FUS-mutant cells. Moreover, we verified the direct impact of mutant FUS by analyzing the expression of select lncRNAs in mutation-corrected isogenic iPSC lines. Here, we will review what is known about the differentially expressed mRNA targets and discuss how their dysfunction may contribute to the development of FUS-linked neurodegenerative disease.

Our study identified two differentially expressed targets associated with transcriptional regulation: LMO3 and ZNF404. LMO3 (LIM domain only 3) is a neuronal basic helix-loop-helix transcriptional regulator involved in cell fate determination and differentiation during embryonic development^26^. LMO3 plays a role in neuronal differentiation of dopaminergic neurons of the substantia nigra, neurons of the globus pallidus externus and has been utilized as a marker of interneuron development^27–30^. LMO3 is specifically involved in the development of dopaminergic neurons by acting as a transcriptional co-activator of Pitx3, ALDH1A1, in addition to genes required for retinoic acid and GABA synthesis ^27^. LMO3 is likewise preferentially expressed in the substantia nigra medial dopaminergic neurons^28^ where its downregulation has been associated with Parkinson’s Disease neurodegeneration^29^. LMO3 expression is also maintained in motor neurons and was shown to be downregulated and alternatively spliced in SHSY cell lines expressing ALS-associated G93A-SOD1 mutations and in SHSY cell lines treated with neurotoxic pesticide Paraquat^31^. Loss of LMO3 was also shown to induce adoption of depressive and anxiety-like behavioral phenotypes in LMO3 knock out mouse models, and was shown to alter animal response to ethanol administration^32^. Finally, LMO3 has been implicated in neuroblastoma progression, where overexpression of LMO3 caused rapid and aggressive tumor growth and was subsequently associated with decreased patient survival^33^. ZNF404 was another differentially expressed gene associated with mutant FUS. ZNF404 is a nuclear zinc finger protein that is predicted to be involved in negative regulation of RNA polymerase II transcriptional activity in addition to other cellular processes, such as DNA binding, and protein-protein interactions^34^. Mutations in ZNF404 have also been recognized in transcriptional analyses of breast cancer and as a regulating factor of gingival progenitor cells^35,36^. The role of ZNF404 in in neural development and neurodegeneration remains unknown. However, given the central role of altered transcriptional regulation and protein-protein interaction in the pathogenesis of ALS, it seems plausible that ZNF404 may contribute to disease progression, possibly through nonspecific dysregulation of entire gene networks. Taken together, our data indicate that FUS may indirectly regulate LMO3 and ZNF404 functionality via lncRNAs. These observations expand what is known regarding the mechanisms of how FUS regulates transcriptional activity. Within the context of ALS, FUS mutant induced loss of LMO3 likely plays a role in the degeneration of motor neurons while increases in lnc-ZNF404, with subsequent downregulation of ZNF404 mRNA, exacerbates wide-scale perturbations in diseased motor neuron transcriptomes. It is important to note, however, the mechanisms underlying these FUS induced alterations persist from iPSCs to eventually affecting motor neurons specifically. How this specificity is achieved remains unknown, and should be the subject of further study.

CRACD (Capping protein inhibiting regulator of Actin Dynamics) is involved in the negative regulation of actin filament capping within the cytosol. This process occurs through direct interactions with actin-capping proteins, resulting in a decreased affinity for actin. This negative regulation prevents the addition of a protective cap onto the barbed end of actin filaments, thereby enabling filament elongation or degradation^37^. It is known that actin filament regulation is critical for neurons as it regulates growth, axon stability, and synaptic function^38–40^. CRACD downregulation has been strongly associated with various types of cancer and metastasis, including small cell lung carcinoma^41^, and colorectal cancer stem cells^42^. Haplotypes of CRACD have been linked to opioid use in patients, as indicated by a recent genome-wide association study (GWAS)^43^. During development, CRACD is expressed during early timepoints and is thought to play a role in tissue differentiation, particularly in the heart and both peripheral and central nervous systems. However, its expression is typically lost in most terminally differentiated adult tissues^44^. Interestingly, our findings show upregulation of CRACD in both iPSCs and terminally differentiated motor neurons. While the exact role of CRACD in neurodegeneration remains unexplored, cytoskeletal dyshomeostasis is a known factor in ALS pathogenesis. For example, mutations in profilin 1 (pfn1), another regulator of actin polymerization, are a known cause of familial ALS^45^. Specifically, it was shown that motor neurons with pfn1 mutations contained decreased levels of pfn1-bound actin with subsequently smaller growth cones and a reduced F-/G-actin ratio, indicating significant cytoskeletal perturbations^45^. Another example is the ALS2 protein, which regulates actin-based neurite outgrowth via the Rab5 GTPase signaling^40,46,47^. These proteins act to regulate actin polymerization and have been associated with early endosome dynamics. Mutations in ALS2 are also a rare cause of juvenile ALS, where the it is believed that it causes unlinking of ALS2 and Rab5 leading to altered actin-based cargo movement and excitatory synaptic signaling^40,48^. Importantly, reports have shown that neurons lacking ALS2 demonstrate greater numbers of glutamate receptors and sensitivity to oxidative stress – both factors which are partially regulated by actin filament dynamics^40,49–54^. Based on these reports, it appears that regulation of actin lies at the crossroads of several different disease-contributing pathways in ALS affected motor neurons. That our data shows upregulation of CRACD suggests mutant FUS may exert currently unknown and potentially significant effects on cytoskeletal homeostasis. Therefore, CRACD involvement in neurodegeneration may prove to be a fruitful avenue for additional investigation.

NR4A2/Nurr1 is a nuclear receptor and transcription factor that has been associated with dopaminergic neuron differentiation, dopaminergic signaling and modulation of microglial and astrocyte mediated inflammation^55^. NR4A2 has been best studied in the context of Parkinson Disease (PD), where its expression levels have been found diminished in both post-mortem tissues and living PD patients^56,57^. NR4A2 has been found to inhibit the expression of proinflammatory mediators and has been linked to a protective role against inflammatory neuron cell death^58^. In fact, knock down of NR4A2 preserved viability of dopaminergic neurons exposed to proinflammatory toxins^59^. While the role of NR4A2 in PD has been best described, more recent reports have demonstrated its role in Alzheimer’s disease, multiple sclerosis, stroke, depression, and intellectual disability^55,60–62^. For example, in rat models of ischemic stroke, NR4A2 was found to be regulated by miR-145–5p, and anti-miR-145–5p treatment enhanced neurological recovery following reperfusion^63^. Interestingly, NR4A2 levels have also been found decreased in post-mortem tissues of aged brains, suggesting NR4A2 may contribute to the aging brain^64^. Another recent report connected NR4A2 to cognitive ability. Specifically, it was found that long lasting changes in synaptic plasticity within the hippocampus are regulated by NR4A2^65^. Based on these documented roles, gene and cell based therapies targeting NR4A2 have become promising candidates for the treatment of neurodegenerative disease^66^. Here, we show that mutant FUS may also contribute to the regulation of NR4A2, possibly via the downregulation of lnc-ERMN. Whether the resulting downregulation of NR4A2 is a compensatory response to cell stress or is actually working in new ways to advance the disease process remains unknown. Taken together, our data add to the growing body of evidence linking NR4A2 to neurodegenerative disease, and highlight two novel means by which disease progression may be targeted via FUS and lnc-ERMN.

GPR149, an orphan G-protein-coupled receptor (GPCR), has a limited documented functional role^67^. Many orphan GPCRs, including GPR149, have been investigated as potential drug targets due to their unknown ligands and functions^68^. Some studies have reported that several orphan GPCRs are highly expressed in the prefrontal cortex of the mouse brain, which is involved in learning and memory^69^. GPR149 is known to regulate myelination and remyelination^70^ and is enriched in oligodendrocyte precursor cells (OPCs) where it negatively regulates OPC to oligodendrocyte differentiation as well as myelination and remyelination. GPR149 deficiency promotes OPC to oligodendrocyte differentiation and earlier myelin development. Blocking GPR149 may even promote myelin repair in demyelinating diseases. GPR149 has also been implicated in neuroendocrine signaling, and was detected in the ventromedial hypothalamus transcriptome of mice where it is highly expressed in inhibitory interneurons. Gene variants of GPR149 have also been reported in studies investigating migraine disorder susceptibility^71,72^. Overall, our data show that cells carrying FUS mutants show an increase in lnc-GPR149 and in its predicted mRNA target, GPR149. These increases in GPR149 are of particular interest given its contribution to inhibitor interneurons and myelination, as excitotoxicity and myelination defects have been noted in some cases of ALS^73,74^. Overall, our findings present a compelling case for further exploration of the role of GPR149 in neurodegeneration.

GO and KEGG enrichment analyses of DEGs indicated that FUS mutations likely affect pathways related to neuronal development and carcinogenesis. These findings suggests that FUS mutations might have broader implications in cellular processes beyond neurodegeneration, especially in light of the fact that FUS itself is an oncogene. Furthermore, IPA and GO network analysis of lncRNA-targeted mRNAs revealed significant biological processes involving RNA metabolism, lncRNA regulation, and DNA damage repair. These results support the idea that FUS mutations contribute to the pathophysiology of neurodegenerative diseases through multiple mechanisms, including the dysregulation of RNA metabolism and impaired DNA repair.

It is important to note that while this study has identified some novel pathways involving lncRNAs in FUS-associated neurodegeneration, additional research will be required to further elucidate how these interactions contribute to neurodegenerative disease. This study has three main limitations. First, the iPSCs utilized in our research may not completely represent the neuronal and glial environment *in vivo*, so future studies using *in vivo* models could offer more comprehensive insights into the molecular repercussions of FUS mutations. Second, our focus was primarily on the impact of FUS mutations on the transcriptional landscape, and further exploration of post-transcriptional and translational effects is essential. Lastly, determining the functional implications of the discovered TAR pairs and their potential contribution to neuronal dysfunction and degeneration will be an important area of future inquiry.

In conclusion, our study provides valuable insights into the molecular mechanisms underlying the pathophysiology of neurodegeneration associated FUS mutations and suggests potential therapeutic targets for the treatment of FUS related neurodegenerative disorders. By investigating the impact of FUS mutations on the transcriptional landscape of iPSCs and their persistence in differentiated motor neurons, our findings contribute to the growing body of knowledge required to develop effective therapies for these devastating disorders. Our study also emphasizes the importance of understanding the role of lncRNAs in modulating gene expression and the intricate interplay between lncRNAs and mRNAs in the context of neurodegenerative disorders. Further research into the functional consequences of the identified TAR pairs and their exact roles in disease pathogenesis will be critical in advancing our understanding of the complex molecular events driving neurodegeneration and in devising targeted therapeutic strategies.

## Methods

### Cell culture

The control human induced pluripotent stem cells (iPSCs) used in this study were obtained from ATCC (#KYOU-DXR0109B). The patient-derived iPSCs and their isogenic controls were previously described^2,75^. All iPSCs were cultured on Geltrex LDEV-Free, hESC-Qualified basement membrane matrix, supplemented with 1X Essential 8 supplement. Colonies were regularly passaged using 0.5 mM EDTA (15575–020, Invitrogen) in Dulbecco’s phosphate-buffered saline (DPBS). The cultures were routinely monitored for mycoplasma contamination by PCR. Motor neurons were generated from the iPSC lines using the previously published protocol^25^.

### RNA isolation, library construction, and sequencing

Approximately 2×10^6^ cells were collected and centrifuged at 4C at 2000rpm for 3 min for extraction of RNA. Total RNA was isolated using Trizol (Thermo Scientific, Waltham, MA, USA) according to manufacturer instruction. RNA purity and quantification was determined using a NanoDrop 2000 spectrophotometer (Thermo Fisher Scientific, Waltham, MA, US). Extracted RNA was sent to BGI (Shenzhen, China) for transcriptome library construction. In this process, RNA quality was measured using Agilent 2100 Bioanalyzer (Agilent Technologies, Santa Clara, CS, US). RNA samples were further purified using Ribo-Zero rRNA Removal Kit before fragmentation. Random primers and TruSeq reverse transcriptase kit were for first-strand cDNA synthesis followed by DNA polymerase I and RNaseH for double-stranded cDNA synthesis. The library was then sequenced using Illumina HiSeq. Low quality reads, adapter contamination, and unknown N bases were filtered. The remaining clean mapped to the reference genome (GRCh38) using HISAT (v2.0.4) with default parameters. StringTie (v1.0.4) was used to create the assembled transcriptome.

### Coding Ability Prediction

Coding ability of the filtered and assembled transcripts was evaluated to distinguish mRNA from lncRNA. Three predictive software tools were utilized to score the coding ability of the transcripts using the same pfam database. Transcripts mapped to pfam were designated mRNA while remaining transcripts were designated lncRNA. Only those transcripts identified by all three tools were utilized for downstream analysis. lncRNA transcripts were annotated using NONCODE database.

### Identification of Differentially Expressed lncRNA and mRNA

lncRNA and mRNA expression levels were calculated using the fragment per kilobase of transcript per million mapped reads (FPKM). To calculate differential expression analysis of genes and transcripts, Bowtie2 was used to align clean reads to the reference sequence and then RSEM was used to calculate gene and transcript expression levels. Subsequently, PossionDis tool (PossionDisFoldChange>=2.00 and FDR<=0.001) was used to analyze the significance of differentially expressed lncRNAs (DELs) and differentially expressed mRNAs (DEGs) between the samples SA (control) and samples SB (FUS-P525L) and SC (FUS-R521H). Initial screening identified DELs and DEGs as those transcripts with p-values <0.05 and absolute log_2_ fold change >=1.5.

### lncRNA target gene prediction

To better understand potential functional roles of DELs, mRNA target gene prediction was used. The analysis methods used in this study included calculating Spearman and Pearson correlation coefficients of expression values of DELs and mRNA. Genes with Spearman_cor>=0.6 and Pearson_cor>=0.6 were selected as interaction pairs. These pairs were then classified as Cis or Trans. Any lncRNA in the 10kB upstream or 20kB downstream of the putative mRNA was designated as cis. Targets beyond this range were identified by binding energy of lncRNA and mRNA using RNAplex.

### Functional Enrichment of DEGs and Target Genes of DELs

Datasets of DEGs, DELs, and DEL targets were cross examined to identify TAR pairs containing DELs predicted to target DEGs; these pairs were further refined such that each DEL and DEG pair exhibited a 5-fold or greater increase/decrease relative to control. These pairs were then selected for further study. Furthermore, datasets of DEGs and DEL target genes were used for functional enrichment analysis using Gene Ontology (GO) analysis, Kyoto Encyclopedia of Genes and Genomes (KEGG) pathway analysis, and Ingenuity Pathway Analysis (IPA - QIAGEN). GO, KEGG and IPA terms with p<0.05 were accepted as significant.

### qRT-PCR validation of differentially expressed lncRNAs and mRNAs

Validity of HiSeq results was determined by RT-PCR using primers selected for certain highly differentially expressed DELs and their DEG targets. The total RNA of each cell line was extracted using Trizol reagent (Invitrogen, Carlsbad, CA, USA), according to the manufacturer’s instruction. Total RNA was reverse transcribed into cDNA using the Super Script Vilo Kit (Thermo Scientific, Waltham, MA, USA). The qRT-PCR amplification was performed in triplicate using the ABI 7500 (Applied Biosystems, Foster City, CA, USA) using the PowerUp SYBER Green Master Mix (Thermo Scientific, Waltham, MA, USA). Multiple housekeeping genes were utilized as internal control and included the HPRT and GAPDH genes. Primers for lncRNAs and mRNAs were purchased from Sigma and are shown in Table S1. The relative expression of each validated gene was determined using the 2^−ΔΔCt^ method. Student’s t-test was performed and results with p<0.05 were accepted as significant.

## Figures and Tables

**Table 1.** Summary of reads after quality control. Raw and filtered reads obtained from Illumina Hi-Seq platform.

Samples	Total Raw Reads	Total Clean Reads	Total Clean Reads Ratio	Total Mapping Reads	Uniquely Mapping Reads
Control	106,975,508	99,081,102	92.620%	92.25%	80.16%
FUS R521H	108,244,484	99,842,418	92.238%	93.48%	80.24%
FUS P525L	92,527,778	85,427,768	92.327%	94.21%	80.90%

**Table 2.** Summary of identified mRNA and lncRNA targets in human iPS cells. Novel mRNAs identified as reads without matches to any major database. Novel lncRNAs identified as reads without matches to NONCODE database.

Sample	Known lncRNA	Known mRNA	Novel lncRNA	Novel mRNA
Control	11,399	15,274	1703	4035
FUS R521H	11,516	15,177	1716	4033
FUS P525L	11,098	15,163	1697	4034
